# Sex frequency and sex planning among men who have sex with men in Bangkok, Thailand: implications for pre- and post-exposure prophylaxis against HIV infection

**DOI:** 10.1186/1758-2652-13-13

**Published:** 2010-04-14

**Authors:** Frits van Griensven, Warunee Thienkrua, Wichuda Sukwicha, Wipas Wimonsate, Supaporn Chaikummao, Anchalee Varangrat, Philip A Mock

**Affiliations:** 1 Thailand Ministry of Public Health - US Centers for Disease Control and Prevention Collaboration, Nonthaburi, Thailand; 2 Division of HIV/AIDS Prevention, Centers for Disease Control and Prevention, Atlanta, Georgia, USA

## Abstract

**Background:**

Daily HIV antiretroviral pre-exposure prophylaxis (PrEP) is being evaluated in clinical trials among men who have sex with men (MSM). However, daily PrEP may not be congruent with sexual exposure profiles of MSM. Here, we investigate sex frequency and sex planning to identify and inform appropriate PrEP strategies for MSM.

**Methods:**

We evaluated sex frequency and sex planning in a cohort of HIV-negative MSM in Bangkok, Thailand. Chi-squared test was used to compare reports of sex on different weekdays; logistic regression was used to identify predictors of sex frequency and sex planning.

**Results:**

Of 823 MSM (with a mean age of 28.3 years), 86% reported having sex on two days per week or less, and 65% reported their last sex to have been planned. Sex on the weekend (~30%) was more often reported than sex on weekdays (~23%). In multivariate analysis, use of alcohol, erectile dysfunction drugs, group sex, sex with a foreigner, buying and selling sex, and a history of HIV testing were associated with having sex on three days or more per week. Being aged 22 to 29 years, not identifying as homosexual, having receptive anal intercourse, and not engaging in group sex were associated with unplanned sex.

**Conclusions:**

Intermittently dosed PrEP (as opposed to daily) may be a feasible HIV prevention strategy and should be considered for evaluation in clinical trials. Risk factors for sex frequency and sex planning may help to identify those in need for daily PrEP and those who may not be able to take a timely pre-exposure dose.

## Background

Despite high levels of knowledge and awareness about the protective efficacy of condom use against HIV infection, the HIV incidence among men who have sex with men (MSM) remains high. Cohort studies among MSM in the western world consistently show an HIV incidence of 1% to 2% per year [1]. Similar studies in the developing world show an HIV incidence of 5% to 8% per year [2,3]. Additional strategies to prevent HIV infection among MSM are therefore urgently needed.

Antiretroviral prophylaxis is widely used to prevent HIV transmission from mother to child, and has been shown to prevent HIV acquisition in health care workers following occupational exposure [4]. Consequently, antiretroviral prophylaxis has been proposed as an experimental strategy to prevent sexual and parenteral transmission of HIV infection in humans [5-7]. Studies of daily pre-exposure prophylaxis (PrEP) and intermittent antiretroviral pre-exposure prophylaxis (iPrEP) with tenofovir (TDF) or Truvada (a combination of TDF and emtricitabine) in non-human primates have shown a significantly reduced risk of simian HIV (SHIV) infection following rectal challenge [8-10].

TDF and Truvada are licensed for the treatment of HIV infection and are generally well tolerated with few side effects. TDF and Truvada are currently tested in Phase III safety and efficacy trials of daily PrEP in injection drug users, high-risk heterosexuals and MSM. A safety study of daily TDF is being conducted simultaneously among MSM in the United States [7,11]. Until these trials are completed, the efficacy of any PrEP regimens, including iPrEP, in preventing HIV infection is unknown.

Even though daily PrEP with Truvada may show efficacy in preventing HIV infection in MSM, we do not know whether daily intake of these drugs is required to prevent infection. Given the long intracellular half-life of TDF, antiretroviral activity may last for several days. Recent animal studies have shown that intermittent pre- and post-exposure prophylaxis with Truvada was efficacious in preventing SHIV infection following rectal exposure [10]. Promising iPrEP regimens tested in these studies included doses at two hours before and 22 hours after exposure, 22 hours before and two hours after, and 72 hours before and two hours after [10].

Daily use of other prophylactic drugs, such as for malaria and tuberculosis, has not been very successful. Daily dosing of Truvada may be associated with toxicity and mild side effects, such as cramping, diarrhea and flatulence, which may interfere with adherence and thus with protection. In addition, MSM may not be exposed to HIV on a daily basis, unlike injection drug users or brothel-based sex workers, and may therefore not be motivated to take drugs daily.

Other prevention modalities for MSM, such as condom use and post-exposure prophylaxis, are exposure driven and intermittent. Anecdotal information suggests that some high-risk MSM are using PrEP intermittently, sometimes provided by their physicians. The available literature, however, suggests that this is an extremely rare phenomenon [12,13].

In addition, the costs of daily PrEP may be financially prohibitive, particularly for MSM in lower- and middle-income countries. A recent analysis estimated that to reduce the lifetime HIV infection risk for a gay man in the United States from 44% to 25%, the per person cost of daily PrEP was $151,600, on top of the $81,100 per person already spent for regular prevention [14]. In a separate analysis, the cost associated with a five-year daily prophylaxis programme for 15,000 MSM in the United States was estimated at $900 million [15].

There are currently no studies evaluating the feasibility of iPrEP for the prevention of HIV infection in MSM. For example, we do not know how often MSM have sex and are potentially exposed to HIV. If most MSM are exposed on a daily basis, iPrEP is not a feasible prevention strategy. It is also unknown to what extent MSM are planning their sex. If the iPrEP regimen consists of a pre- and post-exposure dose, MSM need to know when they are going to have sex, in order to timely administer their pre-exposure dose. Therefore, in this paper we report on the sex frequency, sex planning and their predictive factors in a cohort of HIV-negative MSM in Bangkok, Thailand.

## Methods

### Study population

Between April 2006 and December 2007, 1002 HIV-negative men enrolled in the Bangkok MSM Cohort Study, a prospective study of HIV incidence, follow-up rates and willingness to participate in HIV prevention trials. Men were Thai nationals, residents of Bangkok, at least 18 years old and had had penetrative sex with another man in the past six months. At baseline, demographic and behavioural data were collected using an audio-computer-assisted self-interview.

In the cohort study, men were seen every four months for HIV testing, medical history and behavioural assessment for a duration of three years. Between February 2008 and May 2009, a cross-sectional substudy was conducted in which 823 HIV-negative men completed an audio-computer-assisted self-interview with questions about how often they had sex and how often their sex was planned.

The remaining 179 men (17.9%) were either lost to follow up (n = 150) or seroconverted for HIV infection (n = 29) prior to taking the interview. Men who were lost to follow up were significantly younger and less educated than those who were retained in the study. Since HIV-positive men are not eligible for PrEP, men who seroconverted for HIV infection were not included in this analysis.

### Measures

Questions in the interview included: (1) On the average, how often do you have sex? (answering categories: every day, several times a week, once a week, several times a month, less than once a month); (2) On how many days did you have sex in the past week? (0-7 days); (3) On what days of the past week did you have sex? (Monday, Tuesday ... Sunday); and (4) Was the first sexual encounter on each of those days planned or not? (yes/no). If no sex in the past week was reported, participants were asked whether the first sexual encounter on the last day they had had sex was planned. "Planned sex" was defined as "having made intentional arrangements to have sex, e.g., you went to the park, sauna, bar or online to find a sex partner or you had made an appointment with another man to have sex".

### Statistical analysis

Descriptive statistics were used to characterize sex frequency and sex planning; χ^2 ^was used to compare the proportion of respondents reporting sex on different days of the week, while adjusting for multiple comparisons. Odds ratios and 95% confidence intervals were used to evaluate the association between demographic (at baseline) and behavioural variables (during the four months preceding the interview) and sex frequency and sex planning. Factors independently associated with sex frequency and sex planning were identified by entering variables with P-values of less than 0.05 into backward stepwise logistic regression analysis (SPSS 17.0, SPSS Inc. Chicago, IL, 2008).

For the purpose of this analysis, sex frequency ("on how many days in the past week did you have sex?") was dichotomized into sex on two days per week or less and sex on three days per week or more; sex planning was dichotomized into planned and unplanned based on whether the first sex on the last day the participant reported sex was planned or not. These cut-off points were chosen based on their implications for iPrEP: if men have sex on three days per week or more, the number of pre- and post exposure doses will be almost equal or exceed the number needed for daily PrEP. Hence daily PrEP will be a more appropriate regimen for them; planning of the first sex on a day is necessary for a timely administration of the pre-exposure dose. To retain all subjects in the analysis, sex on the last day was chosen as the dependent variable over a composite measure of planned sex in the past week (since not all men reported sex in the past week).

### Ethical approval

The protocol of the current study was reviewed and approved by the Ethical Review Committee of the Thailand Ministry of Public Health and by an Institutional Review Board of the US Centers for Disease Control and Prevention.

## Results

### Demographic and behavioural characteristics

The mean age of participants was 28.3 years (median 27 years, range 19-58 years), 50.4% had university education, and 59.4% lived away from their families (Table 1). During the four months prior to enrolment, drug use was reported by 8.9% and use of nitrate inhalers by 4.1%; no participants reported to have injected drugs. Use of erectile dysfunction drugs (ever) was reported by 5.3%. The majority of participants (79.2%) identified as homosexual and 37.7% said they were usually the insertive partner in anal sex. Steady male sexual partner(s) were reported by 68.6% and male casual sexual partner(s) by 69.1%; always using a condom with male partners was reported by 43.7%.

**Table 1:** Bivariate and multivariate analysis of having sex on three days or more per week among men who have sex with men in Bangkok, Thailand, 2009

							Bivariate			Multivariate	
Demographics		Total	%	Sex ≥ 3 days	%	OR	95% CI	p	OR	95% CI	p
**Total**		823	100	117	14.2						

**Age group (years)**											

18-21		71	8.6	12	16.9	1.18	0.58-2.39	0.78			

22-29		473	57.5	64	13.5	0.91	0.59-1.39	0.74			

≥ 30		279	33.9	41	14.7	1					

**Employment**											

Yes		625	75.9	90	14.4	1.06	0.67-1.69	0.88			

No		198	24.1	27	13.6	1					

**Education**											

Less than high school		56	6.8	6	10.7	1					

High school or equivalent		352	42.8	58	16.5	1.64	0.67-4.01	0.37			

University and above		415	50.4	53	12.8	1.22	0.49-2.98	0.82			

**Current living situation**											

Live with parents/relatives		334	40.6	37	11.1	1					

Live with partner		104	12.6	19	18.3	1.79	0.98-3.28	0.08			

Live alone/friend/other		385	46.8	61	15.8	1.51	0.98-2.34	0.08			

**Use of alcohol** **(past 4 m)**											

Yes		660	80.2	107	16.2	2.96	1.51-5.80	0.001	2.49	1.24-4.98	0.01

No		163	19.6	10	6.1	1			1		

**Binge drinking** (past 4 m)*(n = 660)											

Yes		76	11.5	17	22.4	1.58	0.88-2.84	0.17			

No		584	88.5	90	15.4	1					

**Use of any drugs** **(past 4 m)**											

Yes		73	8.9	19	26.0	2.34	1.33-4.12	0.002		-^†^	

No		750	91.1	98	13.1	1					

**Inhaled nitrites** **(poppers) (past 4 m)**											

Yes		34	4.1	9	26.5	2.27	1.03-4.99	0.04		-^†^	

No		789	95.9	108	13.7	1					

**Used club drugs^‡^** **(past 4 m)**											

Yes		58	7.0	16	27.6	2.50	1.36-4.62	0.002		-^†^	

No		765	93.0	101	13.2	1					

**Used erectile dysfunction drugs (ever)**											

Yes		43	5.2	18	41.9	4.95	2.61-9.41	<0.001	2.68	1.29-5.55	0.008

No		780	94.8	99	12.7	1			1		

**Sexual self identification (baseline)**											

Homosexual		652	79.2	93	14.3	1.02	0.63-1.65	0.94			

Heterosexual/bisexual/Transgender		171	20.8	24	14.0	1					

**Age at first anal sex (n = 812)**											

≤ 15		115	14.2	21	18.3	1.40	0.83-2.35	0.26			

> 15		697	85.8	96	13.8	1					

**Had steady male partner (past 4 m)**											

Yes		563	68.4	89	15.8	1.56	0.99-2.45	0.07			

No		260	31.6	28	10.8	1					

**Had casual male partner (past 4 m)**											

Yes		568	69.0	95	16.7	2.13	1.30-3.47	0.002			

No		255	31.0	22	8.6	1					

**Usual anal sex position with male (those who had anal sex) (n = 812)**											

Insertive		306	37.7	46	15.0	1.08	0.73-1.62	0.69			

Receptive/both		506	62.3	71	14.0	1					

**Condom use with steady/casual male partners (past 4 m) (n = 771)**											

Always		435	56.4	58	13.3	1					

Not always		336	43.6	57	17.0	1.33	0.89-1.97	0.19			

**Last sex was planned (n = 818)**											

Yes		534	65.3	79	14.8	1.12	0.74-1.71	0.58			

No		284	34.7	38	13.4	1					

**Group sex (ever)**											

Yes		137	16.6	42	30.7	3.60	2.33-5.57	<0.001	2.07	1.27-3.36	0.003

No		686	83.4	75	10.9	1			1		

**Sex with foreigner** **(past 4 m)**											

Yes		115	14.0	34	29.6	3.16	1.99-5.01	<0.001	1.84	1.11-3.06	0.02

No		708	86.0	83	11.7	1			1		

**Coerced into sex (ever)**											

Yes		144	17.5	20	13.9	1					

No		679	82.5	97	14.3	1.03	0.61-1.74	0.90			

**Paid male partner for sex (past 4 m)**											

Yes		96	11.7	25	26.0	2.43	1.47-4.03	<0.001	1.91	1.10-3.30	0.02

No		727	88.3	92	12.7	1			1		

**Condom use with male partner you paid** **(past 4 m) (n = 96)**											

Always		68	70.8	18	26.5	1.08	0.39-2.97	0.88			

Not always		28	29.2	7	25.0	1					

**Got paid for sex by male partner (past 4 m)**											

Yes		97	11.8	33	34.0	3.94	2.44-6.35	<0.001	2.68	1.59-4.52	<0.001

No		726	88.2	84	11.6	1			1		

**Condom use with male partner who paid you (past 4 m) (n = 97)**											

Always		77	79.4	28	36.4	1.71	0.56-5.21	0.49			

Not always		20	20.6	5	25.0	1					

**Suicidal ideation^§ ^(ever)**											

Yes		213	25.9	38	17.8	1.46	0.96-2.23	0.08			

No		610	74.1	79	13.0	1					

**HIV testing (ever)**											

Yes		426	51.8	73	17.1	1.66	1.11-2.48	0.01	1.54	1.001-2.36	0.049

No		397	48.2	44	11.1	1			1		

**Worried about getting HIV infection**											

Never to regularly		587	71.3	77	13.1	1					

Often to always		236	28.7	40	16.9	1.35	0.89-2.05	0.19			

**Worried about getting STI**											

Never to regularly		609	74.0	86	14.1	1					

Often to always		214	26.0	31	14.5	1.03	0.66-1.61	0.99			

**Increased sexual risk behaviour after hearing about effective treatment for HIV infection**											

Not at all to moderate		645	78.4	97	15.0	1.40	0.84-2.34	0.24			

Much to a great deal		178	21.6	20	11.2	1					

*Got drunk 2-3 times per week or more in the past four months among those who used alcohol

^†^Dropped out of the multivariate model

^‡^Club drugs = marijuana, ecstasy, methamphetamine, ketamine, cocaine, and gamma hydroxy butyrate (GHB)

^§^Ever had seriously thought about or attempted suicide

M: month: STI: sexually transmitted infection

### Descriptive analysis of sex frequency and sex planning

**Figure 1:**
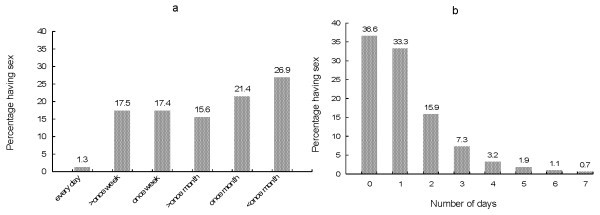
**Frequency of sex (a) and number of days in the past week on which sex was reported (b) among men who have sex with men in Bangkok, Thailand**.

**Figure 2:**
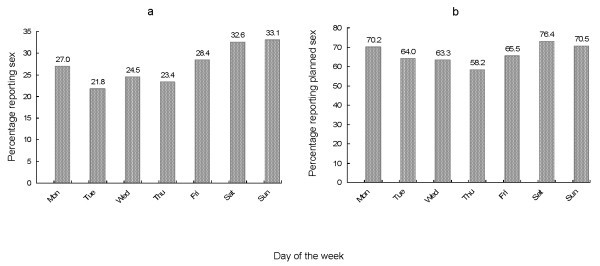
**Days on which sex was reported in the past week (a) and planned first sex on a day (b) among men who have sex with men in Bangkok, Thailand**.

When asked how many times men had sex on average, 1.3% said every day, 17.5% said several times a week and the remaining 81.2% said once a week or less (Figure 1a). When asked about how many days they had had sex in the past week, 85.8% reported having had sex on two days or less, and 14.2% reported having had sex on three days or more (Figure 1b). Of the 522 men who reported sex in the past week: 27.0% reported having it on Monday; ~23.0% on Tuesday, Wednesday or Thursday; 28.4% on Friday; 32.6% on Saturday; and 33.1% Sunday (Figure 2a). Sex on Saturday and Sunday was more often reported than sex on Tuesday, Wednesday or Thursday (p < 0.001).

When asked whether the first sex on each of those days was planned or unplanned, approximately 70% of first sex on Saturday, Sunday and Monday was planned, whereas 60% to 65% of first sex on other weekdays was planned (Figure 2b). There were no significant differences between the percentages of planned sex on different days of the week. During the past week, participants reported 1302 days on which they had had sex, of which the first sex on 860 days (66.1%) was planned. When all participants (including those who did not report having sex in the past week) were asked whether the first sex on the most recent day they had sex was planned, 65.3% answered affirmative.

### Bivariate and multivariate analysis of sex frequency and sex planning

In bivariate analysis of sex frequency, use of alcohol, drugs, erectile dysfunction drugs, having had casual partner(s), group sex, sex with a foreigner, buying and selling sex, and a history of HIV testing were associated with having sex on three or more days of the past week. Demographic factors, other sexual variables, and condom use were not associated with sex frequency (Table 1). In multivariate analysis, use of alcohol, erectile dysfunction drugs, group sex, sex with a foreigner, buying and selling sex, and a history of HIV testing were significantly and independently associated with having sex on three or more days of the week (Table 1).

In bivariate analysis of sex planning, being 22 to 29 years old, being unemployed, having lower education, binge drinking, not identifying as homosexual, being at a younger age at first anal sex, having receptive anal intercourse, having sex once a month or less, and not engaging in group sex were associated with unplanned sex (Table 2). In multivariate analysis, being at a younger age, not identifying as homosexual, having receptive anal intercourse, and not engaging in group sex were significantly and independently associated with unplanned sex (Table 2).

**Table 2:** Bivariate and multivariate analysis of unplanned sex among men who have sex with men in Bangkok, Thailand, 2009

							Bivariate			Multivariate	
Demographics		Total	%	Unplan-ned sex	%	OR	95% CI	p	OR	95% CI	p
**Total**		818	100	284	34.7						

**Age group (years)**											

18-21		70	8.6	32	45.7	2.41	1.40-4.14	0.002	1.71	0.89-3.28	0.11

22-29		470	57.5	180	38.3	1.78	1.28-2.46	0.001	1.67	1.12-2.48	0.01

≥30		278	34.0	72	25.9	1			1		

**Employment**											

Yes		621	75.9	203	32.7	1					

No		197	24.1	81	41.1	1.44	1.03-1.99	0.04		-^†^	

**Education**											

Less than high school		56	6.8	31	55.4	3.13	1.77-5.52	<0.001	2.97	1.51-5.83	0.002

High school or equivalent		350	42.8	136	38.9	1.60	1.18-2.17	0.002	1.36	0.93-1.98	0.11

University and above		412	50.4	117	28.4	1					

**Current living situation**											

Live with parents/relatives		334	40.8	107	32.0	1					

Live with partner		103	12.6	39	37.9	1.29	0.82-2.05	0.33			

Live alone/friend/other		381	46.6	138	36.2	1.20	0.88-1.64	0.27			

**Use of alcohol** **(past 4 m)**											

Yes		657	80.3	239	36.4	1.47	1.01-2.15	0.06			

No		161	19.7	45	28.0	1					

**Binge drinking** (past 4 m)*(n = 657)											

Yes		76	11.6	36	47.4	1.68	1.03-2.71	0.046	1.67	0.99-2.81	0.054

No		581	88.4	203	34.9	1			1		

**Use of any drugs** **(past 4 m)**											

Yes		73	8.9	28	38.4	1.19	0.72-1.95	0.58			

No		745	91.1	256	34.4	1					

**Inhaled nitrates (poppers) (past 4 m)**											

Yes		34	4.2	8	23.5	1					

No		784	95.8	276	35.2	1.77	0.79-3.95	0.22			

**Used club drugs^‡ ^(past 4 m)**											

Yes		58	7.1	24	41.4	1.36	0.79-2.34	0.34			

No		760	92.9	260	34.2	1					

**Used erectile dysfunction drugs (ever)**											

Yes		43	5.3	12	27.9	1					

No		775	94.7	272	35.1	1.40	0.71-2.76	0.42			

**Sexual self-identification (baseline)**											

Homosexual		648	79.2	213	32.9	1			1		

Heterosexual/bisexual/transgender		170	20.8	71	41.8	1.46	1.04-2.07	0.04	1.58	1.04-2.38	0.03

**Age at first anal sex (n = 807)**											

≤ 15		114	14.1	51	44.7	1.63	1.09-2.43	0.02		-^†^	

> 15		693	85.9	230	33.2	1					

**Had steady male partner (past 4 m)**											

Yes		561	68.6	196	34.9	1.03	0.76-1.41	0.91			

No		257	31.4	88	34.2	1					

**Had casual male partner (past 4 m)**											

Yes		565	69.1	191	33.8	0.88	0.65-1.20	0.46			

No		253	30.9	93	36.8	1					

**Usual anal sex position with male (those who had anal sex) (n = 807)**											

Insertive		306	37.9	87	28.4	1			1		

Receptive/both		501	62.1	194	38.7	1.59	1.17-2.16	0.004	1.67	1.16-2.40	0.006

**Condom use with steady/casual male partners (past 4 m) (n = 768)**											

Always		433	56.4	140	32.3	1					

Not always		335	43.6	127	37.9	1.28	0.95-1.72	0.12			

**Frequency of sex**											

Every day/several times per week		155	18.9	45	29.0	1					

Once a week/several times per month		271	33.1	84	31.0	1.10	0.71-1.69	0.75		-^†^	

Once a month or less		392	47.9	155	39.5	1.60	1.07-2.39	0.03			

**Group sex (ever)**											

Yes		137	16.7	34	24.8	1			1		

No		681	83.3	250	36.7	1.76	1.16-2.67	0.01	1.84	1.17-2.91	0.009

**Sex with foreigner** **(past 4 m)**											

Yes		115	14.1	35	30.4	1					

No		703	85.9	249	35.4	1.25	0.82-1.92	0.35			

**Coerced into sex (ever)**											

Yes		114	17.6	60	41.7	1.43	0.99-2.07	0.07			

No		674	82.4	224	33.2	1					

**Paid male partner for sex (past 4 m)**											

Yes		96	11.7	33	34.4	1					

No		722	88.3	251	34.8	1.02	0.65-1.59	1.00			

**Condom use with male partner you paid** **(past 4 m) (n = 96)**											

Always		68	70.8	22	32.4	1					

Not always		28	29.2	11	39.3	1.35	0.54-3.37	0.68			

**Got paid for sex by male partner (past 4 m)**											

Yes		96	11.7	36	37.5	1.15	0.74-1.78	0.62			

No		722	88.3	248	34.3	1					

**Condom use with male partner who paid you (past 4 m) (n = 96)**											

Always		76	79.2	27	35.5	1					

Not always		20	20.8	9	45.5	1.48	0.55-4.03	0.60			

**Suicidal ideation^§ ^(ever)**											

Yes		212	25.9	84	39.6	1.33	0.96-1.84	0.10			

No		606	74.1	200	33.0	1					

**HIV testing (ever)**											

Yes		425	52.0	140	32.9	1					

No		393	48.0	144	36.9	1.18	0.88-1.57	0.30			

**Worried about getting HIV infection**											

Never to regularly		583	71.3	205	35.2	1.07	0.78-1.47	0.73			

Often to always		235	28.7	79	33.6	1					

**Worried about getting STI**											

Never to regularly		605	74.0	211	34.9	1.03	0.74-1.43	0.94			

Often to always		213	26.0	73	34.3	1					

**Increase sexual risk behaviour after hearing about effective treatment for HIV infection**											

Not at all to moderate		641	78.4	214	33.4	1					

Much to a great deal		177	21.6	70	39.5	1.30	0.93-1.84	0.15			

*Got drunk 2-3 times per week or more in the past four months among those who used alcohol

^†^Dropped out of the multivariate model

^‡^Club drugs = marijuana, ecstasy, methamphetamine, ketamine, cocaine, and gamma hydroxy butyrate (GHB)

^§^Ever had seriously thought about or attempted suicide

M: month: STI: sexually transmitted infection

## Discussion

Most of the MSM enrolled in this study were exposed to possible HIV infection only intermittently, and about two-thirds of MSM had a window of opportunity to take a pre-exposure dose of chemoprophylaxis prior to sexual activity. This information implies that in terms of frequency of exposure, iPrEP may be a more appropriate regimen than daily PrEP. Unfortunately, those lacking the opportunity to take a timely pre-exposure dose were also at high behavioural risk for HIV infection.

Factors in favour of iPrEP over daily PrEP include reduced costs, decreased pill burden, and possibly, reduced toxicity and drug side effects. Cost-effectiveness analysis has shown that implementation of daily PrEP would be extremely challenging, even in the developed world [14,15]. In the developing world, where most of the infections occur, daily PrEP would generally be not affordable.

With respect to pill burden, daily intake of drugs for considerable time in the absence of overt disease has proven to be difficult for many people. Toxicity, in particular renal toxicity, and dysfunction have been reported in association with TDF treatment [16]. Fewer doses of drugs may reduce mild side effects, such as cramping, diarrhea and flatulence, and may therefore positively affect adherence.

As expected, sex was significantly more often reported on the weekend, but differences with other days were small. For those who almost exclusively have sex on the weekend, this may provide an opportunity to develop pill-taking routines, which may help to improve adherence.

With regard to sex planning, there were no significant differences between the different days of the week. With 60% to 65% of the first sex on a day being planned, the majority of men would principally be able to take pre-exposure doses, but still, about one-third of men would not. For these men, a regimen consisting of a number of weekly standing doses followed by post-exposure doses may be more appropriate in terms of opportunities for adherence. We need to keep in mind, however, that in these analyses, sex planning is used as a proxy for pill-taking behaviour, and that we do not know whether those who plan their sex will also take their pre-exposure doses as indicated.

Factors in our assessment that were independently and significantly associated with more frequent sex were use of alcohol, erectile dysfunction drugs, group sex, sex with a foreigner, buying and selling sex, and a history of HIV testing. Use of erectile dysfunction drugs, group sex, sex with a foreigner, buying and selling sex, and a history of HIV testing may be indicators of a more active sex life, while alcohol use may be associated with decreased control over one's sexual behavior. Together, these factors may help to identify those who should be targeted for daily PrEP.

Being at a younger age, not identifying as homosexual, having receptive anal intercourse, and not engaging in group sex were independently and significantly associated with unplanned sex. Those who are younger and those not engaging in group sex may be less likely or able to plan their sex due to lack of experience or decreased control over their sexual desire. Lack of control over partner selection or the necessity to respond to sexual opportunities may explain the association between not identifying as homosexual, receptive anal intercourse, and unplanned sex.

The association between younger age, receptive anal intercourse and unplanned sex is problematic, since the former two are also known as risk factors for HIV infection [17]. Alternative PrEP regimens, such as two standing doses per week plus a post-exposure dose, if proven efficacious, or other regimens or prevention methods, may therefore be necessary for those who have unplanned sex.

The results presented in this paper are subject to a number of limitations. First of all, our data were collected in a sample of men at high risk for HIV infection who may not be representative of all MSM. Men at lower risk may have different sex frequency and sex planning profiles. In addition, younger and less educated men were more likely to have been lost to follow up in the current study. Since both younger age and lower education were found associated with unplanned sex, absence of these men may have biased our results regarding sex planning upwards.

Finally, our data are cross sectional and do not provide information about changes in sex frequency and sex planning profiles over time. For the same reason, our risk factor analysis does not allow causal inference; for example, it is unknown whether the risk factors occurred before or after sexual frequency and sex planning profiles were established.

## Conclusions

Sexual behaviour profiles of MSM enrolled in this study indicate that most men were exposed to HIV infection only intermittently, and that most men had a window of opportunity to take a pre-exposure dose prior to sexual activity. This information implies that in terms of sex frequency, iPrEP may be a more appropriate regimen than daily PrEP. Unfortunately, those lacking the opportunity to take a timely pre-exposure dose were at high behavioural risk for HIV infection.

Phase III trials of daily PrEP in humans are still ongoing and it is unknown whether this regimen will be able to prevent infection. iPrEP regimens discussed here (pre- and post-exposure doses at different time intervals) have not yet entered clinical evaluation in humans, but animal studies are promising. Therefore, our data may help to inform iPrEP regimens for evaluation in future clinical trials in MSM.

## Competing interests

The authors declare that they have no competing interests.

## Authors' contributions

FvG designed the study and wrote the first version of the manuscript. WT performed statistical analysis and reviewed the manuscript. WS programmed the questionnaire and managed the data. WW oversaw the recruitment and retention of study participants and performed statistical analysis. SC oversaw the execution of the study and collection of data. AV designed the study questionnaire and critically reviewed the manuscript. PM oversaw database management and statistical analysis. All authors read and approved the final manuscript.

## Disclaimer

The findings and views presented in this paper are those of the authors and do not necessarily represent the views of the US Centers for Disease Control and Prevention. Any use of trade product, or firm names in this publication is for descriptive purposes only and does not imply endorsement by the US Government.
